# SynLlama: Generating Synthesizable Molecules and Their Analogs with Large Language Models

**Published:** 2025-04-18

**Authors:** Kunyang Sun, Dorian Bagni, Joseph M. Cavanagh, Yingze Wang, Jacob M. Sawyer, Andrew Gritsevskiy, Oufan Zhang, Teresa Head-Gordon

**Affiliations:** 1 Kenneth S. Pitzer Theory Center and Department of Chemistry; 2 Department of Bioengineering, University of California, Berkeley, CA, 94720 USA; 3 Department of Chemical and Biomolecular Engineering, University of California, Berkeley, CA, 94720 USA; 4 Department of Chemistry, University of Minnesota, 207 Pleasant Street SE, Minneapolis, MN 55455, USA; 5 Contramont Research, San Francisco, CA, 94158 USA

## Abstract

Generative machine learning models for small molecule drug discovery have shown immense promise, but many molecules they generate are too difficult to synthesize, making them impractical for further investigation or development. In this work, we present a novel approach by fine-tuning Meta’s Llama3 Large Language Models (LLMs) to create SynLlama, which generates full synthetic pathways made of commonly accessible building blocks and robust organic reaction templates. SynLlama explores a large synthesizable space using significantly less data compared to other state-of-the-art methods, and offers strong performance in bottom-up synthesis, synthesizable analog generation, and hit expansion, offering medicinal chemists a valuable tool for drug discovery developments. We find that SynLlama, even without training on external building blocks, can effectively generalize to unseen yet purchasable building blocks, meaning that its reconstruction capabilities extend to a broader synthesizable chemical space than the training data. We also demonstrate the use of SynLlama in a pharmaceutical context for synthesis planning of analog molecules and hit expansion leads for proposed inhibitors of target proteins.

## 1 Introduction

Drug discovery campaigns have led to the development of pharmaceuticals that treat a variety of diseases and have significantly contributed to the increase of human life expectancy over the past several decades.^1–6^ In order to better optimize the drug discovery pipeline for new small molecule therapeutics, early screening steps require the construction of large molecular libraries, such as custom designed DNA-encoded libraries^7^ or the commercially available Enamine libraries^8^, that allow for high-throughput experimental and virtual screening of drug binders to desirable targets. To illustrate, the Enamine REAL library comprises 38 billion drug-like compounds that take advantage of a large set of optimized reactions and building blocks that allow for rapid and reliable synthesis with over 85% success rate. ^8^ DNA-encoded libraries of drug-like molecules^9^, with upper bounds of 100 billion molecules, define a unique DNA sequence that acts as a “bar code” defining its synthesis pathway. Therefore, these extensive libraries enable both experimental and virtual screening methods to identify tightly binding molecules for desired targets.^10–14^ However, despite their size, these libraries often fail to produce active compounds for some drug targets due to insufficient chemical diversity. In fact, the estimated chemical space of drug-like molecules consists of ∼10^60^ compounds, suggesting that even the largest existing screening collections represent only a minuscule fraction of all possible candidates.^15^

With recent advances in artificial intelligence and deep learning, generative models have begun to contribute to these efforts. After training on databases containing various ligand representations^16–20^, machine learning models, including string-based 1D generative models and structure-aware 3D *de novo* methods, have paved the way for quick exploration of greater swaths of unseen chemical space^21–38^. However, even with their exceptional generative capabilities, these models still face one major challenge: their proposed *de novo* molecules lack practical guarantees of synthesizability, which limits their utility to medicinal chemists and drug discovery in general^39–41^. For generative approaches in small-molecule drug discovery to fulfill their potential, ensuring synthetic feasibility is essential to bridge the gap between *in silico* drug design and the realistic applicability of computationally generated molecules.

Efforts have been made to address the problem of poor synthesizability of *de-novo*-generated molecules. One line of research focuses on integrating empirical scoring functions, such as the SA score^42^, into the objective functions of learning algorithms. However, optimizing only the SA score can still lead to generation of unsynthesizable molecules because the scoring function only relies on identifying common fragments in molecules^43^. In addition, these scoring functions also assign bad scores to complex yet synthesizable molecules that require multi-step synthetic pathways, which causes generative models to miss viable candidates^44^.

Alternatively, the proposal of molecular candidates using commercially available building blocks and commonly known organic reaction templates through forward synthesis offers synthetic tractability. Importantly, this strategy is appealing to medicinal chemists, since it offers realistic and actionable synthesis pathways for them to examine and execute. Some models in this direction apply rule-based synthesis and optimization on building blocks or entire synthetic pathways to generate novel molecules with desired chemical properties ^45–47^. Other models condition on input molecules to propose synthetic pathways using commercially available building blocks and well-validated reaction templates. The resulting molecules from proposed syntheses can lead to either the full construction of the target molecule or the generation of structurally similar analogs within the predefined chemical search space. For example, SynNet^48^ constructs synthetic trees via Markov Decision Processes (MDPs) and uses multilayer perceptrons to choose the next action space, but it struggles in planning synthesis for previously unseen molecules. More recent models such as ChemProjector^49^ and Synformer^50^ use transformers to decode for the next action space and have achieved good empirical performances. However, a major drawback of these ML models is their reliance on vast amounts of reaction data (on the order of a hundred million reaction pathways) and a predefined chemical search space dictated by available building blocks and reaction templates. This constraint not only makes training computationally expensive but also limits their ability to propose truly novel synthesis pathways.

Given these challenges, Large Language Models (LLMs) have emerged as a compelling alternative due to their foundational nature and adaptability to downstream tasks.^51^ LLMs inherently possess extensive chemical knowledge, and recent advancements have focused on extracting and applying this knowledge for predictive and optimization tasks using natural language guidance ^52–55^. Furthermore, after some supervised fine-tuning, LLM models can perform at the same level as chemical language models trained solely on chemical representations, all while requiring less data.^38^ The efficiency gained from fine-tuning LLMs thus motivates us to explore their potential in more complex tasks, such as forward synthesis planning, which could pave the way for more cost-effective developments in drug discovery.

Herein, we present SynLlama, an LLM-based tool built on the open-source Llama-3.1–8B and Llama-3.2–1B foundation models ^56^ to deduce synthetic routes for target molecules or structurally related analogs. SynLlama predicts the necessary reaction (RXN) sequences and commercially available building blocks (BBs) required for synthesis, presenting molecules within a diverse yet synthesizable chemical space. Despite being trained on nearly one to two orders of magnitude less data than the current state-of-the-art models, SynLlama demonstrates competitive performance in key tasks for drug discovery, including synthesis planning for target and analog molecules of pharmaceutical interest and hit expansion. Moreover, because of its generative nature, SynLlama has the added ability to explore commercially available building blocks beyond the predefined synthetic space introduced during training - an ability that previous models lack. By integrating molecular design with synthetic feasibility, SynLlama represents a step forward in bridging computational chemistry with synthetic chemistry, providing medicinal chemists with actionable and experimentally accessible molecular candidates.

## 2 Methods

**Figure 1:**
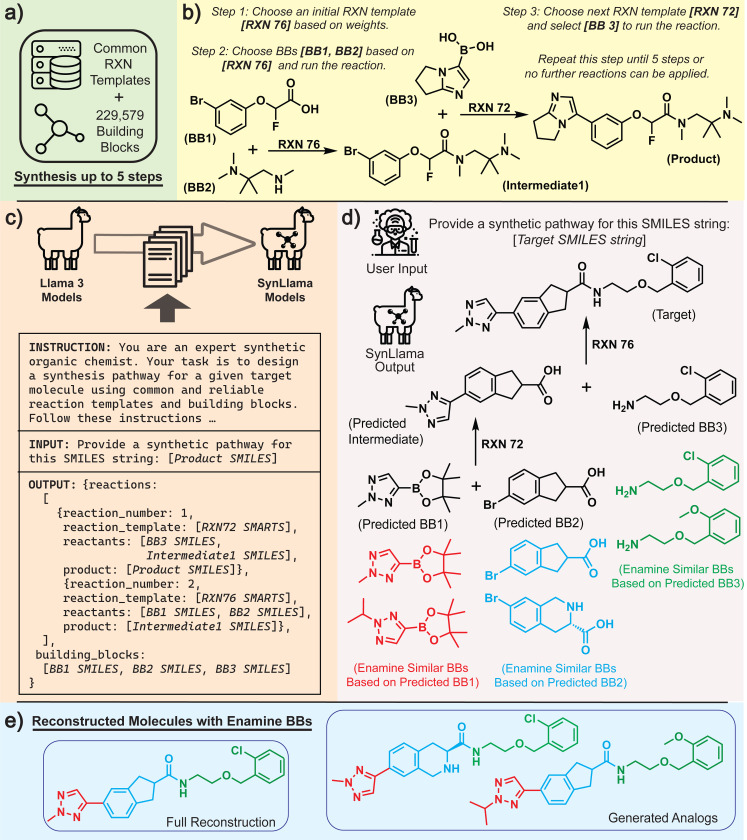
Overview of the SynLlama workflow including data generation, supervised fine-tuning, inference, and reconstruction. (a). The predefined synthesizable chemical space of reaction templates (RXN) and building blocks (BBs) that covers billions of molecules. (b). An example synthesis data and its generation process from the defined synthesizable chemical space to create training examples. Here, RXN 76 represents amide coupling and RXN 72 represents Suzuki coupling. (c). A schematic representation of supervised fine-tuning that converts Llama 3 models to SynLlama models, along with the instruction, input, and output for the example synthesis in (b). (d). SynLlama’s inference on an unseen test molecule. Black represents SynLlama’s raw retrosynthetic output consisting of RXN sequences and predicted BBs, while colored BBs indicate the top two most similar BBs to the predicted ones from the Enamine building block library. Here, RXN 76 represents amide coupling and RXN 72 represents Suzuki coupling. (e). Reconstructed molecules using the predicted reaction sequences and similar building blocks from the Enamine building block library. In this example, all predicted building blocks are present in the Enamine library, allowing for the complete reconstruction of the input molecule and the generation of close analogs.

The SynLlama workflow, illustrated in Figure 1, is designed to generate synthesizable compounds within an expanded chemical space. When an input molecule passes through this workflow, it can either be fully reconstructed through valid synthetic pathways, or the workflow will produce a structurally similar yet synthesizable analog along with its synthesis route. To transform general-purpose LLMs, like the Llama 3 models^56^, into expert models for synthetic pathways, we use three key components: 1) a reliable and diverse set of reaction data that covers a large synthesizable chemical space, 2) an efficient supervised fine-tuning (SFT) strategy to train a general-purpose LLM on these reaction data, and 3) a reconstruction algorithm that can convert the output of the fine-tuned LLM into valid synthesis routes, ensuring the proposed molecules lie within a commercially available chemical search space. These components are crucial for leveraging LLMs, which are known to perform well in diverse chemistry tasks^57,58^, to specialize in synthetic modeling. The following sections will detail the methods and procedures used to develop the SynLlama models, providing a holistic understanding of the entire process.

### 2.1 Reaction Data Generation

As illustrated in Figure 1(a), our defined chemical space for training consists of molecules that can be synthesized in at most five steps with 229,579 Enamine building blocks^20^ (BBs) and 2 sets of well-validated common organic reactions (RXNs). The first set of 91 reactions (RXN Set 1) is selected by Gao et al.^48^ from the works of Hartenfeller et al.^59^ and Button et al.^60^. The second set of 115 reactions (RXN Set 2) is selected by Gao et al.^50^ that contains reactions used to create the Enamine REAL space ^20^ plus some reactions from RXN Set 1. As a result, there are ∼10^30^ molecules within this space that can be represented by a synthesis path that comprises a sequence of BBs and RXNs. To enumerate molecules within this space, we use an iterative approach by selecting RXN templates and searching for compatible BBs. Specifically, as demonstrated in Figure 1(b), the selection of the initial RXN is guided by a probabilistic model based on the number of compatible BBs. Within these compatible BB reactants, the initial BBs are selected at random to form an intermediate via the selected RXN template. This intermediate is then used to match for subsequent RXNs and recruit additional BBs to expand the molecular synthesis pathway. The expansion process continues until no further reactions are possible or the reaction reaches five steps. After training on these representations, the resulting LLM will be able to build powerful connections by mapping input molecules to a sequence of BBs and RXNs that represent synthesis routes.

### 2.2 Supervised Fine-tuning and Inference from SynLlama

To create the SynLlama model, we need to establish data generation protocols for supervised fine-tuning (SFT) of the Llama 3 models as schematically shown in Figure 1(c). When generating reaction data in text format, we choose to represent the BBs and intermediates along the synthetic pathway using SMILES^61^ strings, while RXNs are explicitly defined in the SMARTS^62^ format. These structured chemical notations are designed to enhance SynLlama’s ability to systematically identify and deconstruct bonds according to RXN templates, effectively dismantling input molecules into building-block-sized fragments.

To define our data splits, we apply a time split of the Enamine BBs where we take all BBs from their August 2024 release as the training BBs and all new BBs from their Februray 2025 release that were not in training as the testing BBs. This procedure results in ∼230,000 BBs for training and ∼13,000 for testing, while all reaction templates are accessible to both sets of BBs, thus defining the training and testing chemical spaces. In addition, since our goal is for SynLlama to learn to link molecules with their synthesis routes, our prompt-response pairs are structured according to retrosynthesis, as depicted in Figure 1(c) and shown in detail in Supplementary Figure S1. Such engineered prompts and responses allow the SynLlama model to learn to construct synthesis pathways for the input molecules by inferring sequences of BBs and RXNs, as well as the intermediate steps. While theoretically the model could predict BBs and RXNs without intermediates, we still include them in individual reaction steps in the hope of activating the inherent chemical knowledge in LLMs and enhancing their understanding of synthesis patterns.

We have considered both Llama-3.1–8B (8 Billion parameters) and Llama-3.2–1B (1 Billion parameters) for SFT using datasets of varying sizes. Specifically, Llama-3.1–8B is fine-tuned with datasets containing 100k and 500k synthesis routes, requiring 40 and 240 A-40 hours respectively. Llama-3.2–1B, on the other hand, is trained with datasets containing 500k and 2M synthesis entries, requiring approximately 60 and 240 hours respectively. Herein, we refer to the trained models as SynLlama-(parameter count)-(number of reactions trained) in the first part of Results. For example, SynLlama-1B-2M represents a model fine-tuned from Llama-3.2–1B with 2M synthesis routes. Further details of the SFT are provided in the Supplementary Information.

After training the SynLlama models, we apply the consistent prompt setup to perform inferences on molecules. For any given molecule, the SynLlama models predict reaction sequences in SMARTS format and generate SMILES strings for all the reactants, products, and BBs for the reactions they predict. During inference time, the instruction to SynLlama remains the same, and SMILES strings in the input section are substituted with ones specified by the user. As depicted in Figure S1, the responses of the SynLlama models follow the output structures enforced by the prepared training prompt-response pairs. To be more specific, the output response section consists of two parts: reactions and building blocks. In the ‘reactions’ component, the model sequentially deconstructs the target molecule by breaking bonds using provided reaction templates in a retrosynthetic manner. At each step, it predicts a reaction template, along with the reactants and product of the reaction, continuing until no further reactions are possible. Then, in the ‘building blocks’ section, the model compiles all building blocks, namely, reactants from each reaction that are not products in other reactions, identified from the ‘reaction’ section. A visual representation of the inference process is illustrated in black ink in Figure 1(d).

### 2.3 Reconstruction from Predicted Retrosynthesis

Using the predicted sequence of RXNs and BBs from SynLlama responses, we can synthesize the proposed target molecule or close analogs by applying the predicted reaction templates to the BBs in the inferred order, as shown in black ink in Figure 1(d). In some cases, the predicted BBs match known Enamine BBs, ensuring that the resulting molecules remain within an established chemical space for synthesis. However, due to SynLlama’s generative nature, some predicted BBs are novel. As detailed in Table S2, some of SynLlama’s outputs provide valid synthesis pathways involving at least one BB not found in the Enamine library, but these BBs can be purchased from other suppliers identified by Molport^63^. Therefore, while SynLlama primarily produces molecules within the predefined chemical space using Enamine BBs, its output also offers an alternative strategy for molecule construction. We will revisit this point in the Results section.

When the input molecule cannot be fully reconstructed, we generate analogs by mapping the predicted BBs from SynLlama to known Enamine BBs, thereby sampling molecules from the well-defined Enamine chemical space. Under this scenario, we use nearest neighbor search algorithms with different molecular representations (SMILES and Morgan Fingerprints^64^) to sample Enamine neighboring BBs from the predicted BBs, as illustrated in colored inks in Figure 1(d). Since in SynLlama’s output, the RXN sequences are predicted concurrently with the BBs, our effective search space is constrained to Enamine BBs that can react through the specific RXN template. This smaller Enamine search space not only allows us to ensure the success rate of such forward syntheses but also allows us to effectively explore segments of the input molecule. Further details of the nearest neighbor search algorithms are provided in the Supplementary Information.

When constructing full synthetic pathways for reactions with multiple possible products, we select the product that most closely matches the predicted product based on SMILES string similarity. As shown in Figure 1(e), the reconstruction algorithm iteratively builds synthesis routes, utilizing all predicted BBs and RXN sequences to reconstruct or generate variations of the original molecule from the synthesizable chemical space. This reconstruction algorithm enables the SynLlama model to function as a generator for synthesizable molecules along with their corresponding synthetic pathways.

### 2.4 SynLlama Model Benchmarks

Since we are formulating the synthetic tasks using purely language-based modeling, where all reactions are expressed in SMARTS templates and molecules in SMILES strings, it is extremely important to quantify the capacity of SynLlama models for instruction following and comprehension of reaction chemistry. To assess SynLlama’s ability to follow instructions, we select three benchmarking criteria as shown in Table 1. The first is “Valid JSON,” which examines whether the output format is a parsable JSON following the fine-tuned templates that will be necessary for the downstream reconstruction algorithm. The second criterion is “Template Memorization,” which assesses the model’s ability to memorize the provided reaction templates that define our synthesizable chemical space. Lastly, we benchmark on “BB Selection,” which evaluates whether the “building blocks” section in the responses can accurately identify and select all the building blocks from the “reactions” section of the responses.

To assess SynLlama’s comprehension of reaction chemistry, we focus on individual reactions and summarize the three critical aspects as: (1) the percentage of “Valid SMILES” out of all SMILES strings in the responses, which is essential for assessing SynLlama’s learning outcome of string-based chemical representations in general, (2) the percentage of “Matched Reactants,” which calculates whether the generated reactants match the reactant templates specified in the predicted reactions, and (3) the percentage of “Good Products,” which assess if the predicted product can indeed be generated by applying the proposed reaction templates onto the reactants. Overall, these six benchmarks can collectively assess SynLlama’s capability to follow instructions and perform chemical reactions in string representations.

In Table 1, all four trained SynLlama models are evaluated on both in-distribution training data and out-of-distribution testing and ChEMBL^19^ data to assess the benchmarks outlined above. In the instruction-following benchmarks, most models exhibit strong adherence (over 90 percent) to the fine-tuned response structure across all datasets. This impressive performance indicates that fine-tuning effectively retains the specified output structure when trained with over 100,000 samples. Furthermore, all four models successfully memorized the provided RXN templates and selected the building blocks (BBs) from all predicted reactants over 99 percent of the time. This capability further enhances the coupling effectiveness of the downstream reconstruction algorithm with the SynLlama raw output, as it only requires information about reaction sequences and predicted building blocks.

In the reaction chemistry benchmarking results, a clearer trend emerges: models, regardless of their parameter size, show improved comprehension of reaction chemistry in all three datasets as the amount of training data increases. Notably, most models maintain their performance from training to testing data, but exhibit a greater decline in “Matched Reactants” and “Good Products” performance when generalizing to the ChEMBL data. The reason behind this is that the testing data are generated in the same manner as the training data but with a different set of building blocks, while the ChEMBL data occupies a different chemical space, as previously noted by Gao et al.^48^. Despite the reductions in their performance for ChEMBL molecules, as shown in Supplementary Figure S2, SynLlama-8B-500k and SynLlama-1B-2M can still generate complete and valid syntheses over 50% of the time without any downstream processing. These results indicates that SynLlama’s raw results alone have potential utility for synthesis planning for unseen drug-like molecules.

When comparing SynLlama-8B-500k and SynLlama-1B-500k, we observe that the larger model demonstrates better performance when trained on the same amount of data. Although additional training data could further improve the 8B model based on the current trend, its higher computational cost makes this pursuit less practical. However, as the fine-tuning computational costs for SynLlama-8B-500k and SynLlama-1B-2M require approximately the same A40-GPU hours, and given the comparable benchmark performance between them, we decided to move forward with SynLlama-1B-2M, simplified as SynLlama, for the subsequent tasks due to its faster inference speed.

## 3 Results

### 3.1 Synthesis Planning for Unseen Molecules

Having demonstrated that SynLlama models can reliably predict reaction sequences and building blocks, we now use SynLlama to plan the synthesis of two groups of previously unseen molecules. The first group comprises two sets of 1000 testing molecules, generated similarly to the training data using testing building blocks and corresponding reaction templates. The second group includes two sets of 1000 molecules extracted from the publicly available ChEMBL database^19^ and the Enamine diversity set^20^.

Initially, we follow the standard reconstruction approach within the Enamine chemical space: SynLlama generates raw output predictions in a retrosynthetic manner, and this output then gets processed through the reconstruction algorithm to create target input molecules or analogs using building blocks exclusively from the Enamine library. This evaluation method aligns with that used by existing approaches such as SynNet^48^, ChemProjector^49^, and Synformer^50^, and we compare these methods on the number of reconstructions of the target molecule with Enamine BBs in Table 2. In this comparison, SynNet^48^ serves as a baseline for synthesizable chemical space coverage. ^49^ Furthermore, when trained on their respective reaction sets and using just Enamine BBs along, SynLlama outperforms ChemProjector^49^ and matches the performance of Synformer^50^, while reducing the number of training data by 60-fold and 40-fold.

In addition to reducing data requirements for training, SynLlama can also reconstruct target molecules using commercially available BBs beyond Enamine, even without specific training for this purpose. These ‘New BBs’ add possible synthetic pathways to reconstruct target molecules in all four datasets, as seen in Table 2 and detailed in Tables S3 and S4. Since some target molecules can be synthesized through multiple pathways, either using only Enamine BBs or with the addition of New BBs, the ‘Total’ column in the table reflects the number of unique target molecules reconstructed with SynLlama. With these New BBs, SynLlama’s best reconstruction rates increase to 66.9%, 58.2%, 74.1%, and 28.7% for the 1000 molecules in both Testing Sets, Enamine, and ChEMBL data, respectively. These results clearly outperform previous methods that lack such search capacity of novel building blocks. It is especially noteworthy that New BBs significantly enhance synthetic accessibility of unseen molecules, suggesting that SynLlama learns reaction chemistry well enough to predict novel building blocks.

Finally, when the target molecule cannot be reconstructed, we assess the quality of the analog using three molecular similarity scores between the target molecule and its most similar analog: (1) Tanimoto similarity based on 4096-bit Morgan fingerprints^64^, (2) 4096-bit Morgan fingerprints of Murcko scaffolds^65^, and (3) Gobbi 2D pharmacophore fingerprints^66^. In table 2, the similarity metrics reported are average values of all trial molecules, including those that are fully reconstructed. In 2, we only show the first similarity metric due to the space limit. More details on the metrics and results for molecules that cannot be reconstructed are explored in Tables S5 and S6. Overall, these results collectively show that SynLlama is capable of planning synthesis for unseen molecules or their analogs with purchasable building blocks, highlighting its potential use in drug discovery.

### 3.2 Synthesizable Analog Search for *De Novo* Molecules

Previous research has shown that molecules proposed by generative models often face challenges in practical synthesizability^39–41^, even when optimized using empirical heuristics for synthetic accessibility. In this section, we demonstrate SynLlama’s potential to bridge the gap between generative molecule design and practical synthesis. Specifically, SynLlama is integrated with candidates from a structure-based generative model, iMiner^30^, which optimizes drug-likeness and molecular docking scores against the SARS-CoV-2 Main Protease (Mpro). More details in regards to iMiner are provided in the Supplementary Information.

**Figure 2:**
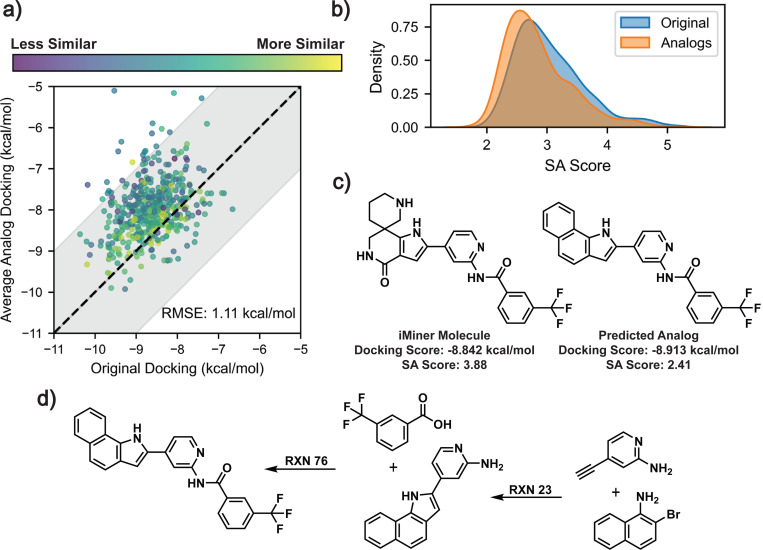
SynLlama performance on generating synthesizable analogs for iMiner proposed binders to SARS-CoV2 Mpro. (a). Correlation plot comparing docking scores of iMiner molecules and the average docking scores of ten most similar analogs (based on Morgan Fingerprints) proposed by SynLlama trained on RXN Set 2. Each data point is color-coded by the average Morgan fingerprint similarity computed between iMiner molecules and analogs, with brighter being more similar and darker being less similar. The shaded area represents an energy uncertainty range of ±2*kcal*/*mol*, which is the typical uncerntainty for AutoDock Vina scores. (b). SA score distribution of iMiner molecules and SynLlama-proposed analogs. (c). Example iMiner molecule and one of its analog proposed by SynLlama. Using the iMiner molecule as input, SynLlama successfully proposes an analog that retains docking score with decreased SA score. For each binder, we average scores from the top 10 structurally similar molecules for a meaningful distributional analysis. (d) Synthesis pathway of the analog molecule of an iMiner *de novo* molecule in (c) using SynLlama BBs via indole synthesis (RXN 23) and amide coupling (RXN 76) reaction templates.

Here, we select the top 500 iMiner-generated binders based on their AutoDock Vina^67^ docking scores to Mpro (PDB: 7L11^68^), and process them through SynLlama, trained on RXN Set 2, to generate synthesizable analogs constrained to Enamine BBs. For the top-10 generated analogs, we perform molecular docking with the same procedure and report their average docking score and average Morgan fingerprint similarity in Figure 2. We choose the SynLlama model trained on RXN Set 2 for their superior performance in analog generation explored in the previous section. A similar distribution analysis of the iMiner analogs generated with SynLlama trained on RXN Set 1 is presented in Figure S3.

In Figure 2(a), the RMSE of docking scores of the generated analogs with respect to the original iMiner molecules is 1.04 kcal/mol, which is an acceptable range of inherent docking score errors reported by Trott et al^67^. Furthermore, as demonstrated in Figure 2(b), the SA score distribution of the generated analogs show a notable decrease from de novo generated binders, demonstrating improved synthetic accessibility of the analogs proposed by SynLlama. These results suggest that SynLlama can generate synthetic pathways for iMiner-proposed molecules or their analogs, enhancing synthetic accessibility of *de novo* molecules when combined with generative models.

### 3.3 Local Hit Expansion for Binder Molecules

Because SynLlama breaks down the original target molecule for synthesis into building blocks, by nature this method allows diverse exploration around parts of the molecular scaffold rather than only on a whole target molecule. In a final task, we apply SynLlama to expand on a hit molecule for the same SARS-CoV-2 Mpro protein (PDB: 7LTJ)^69^ to discover synthesizable molecules that have better relative binding free energies (RBFEs) confirmed by both experiments and accurate free energy perturbation (FEP) calculations.

**Figure 3:**
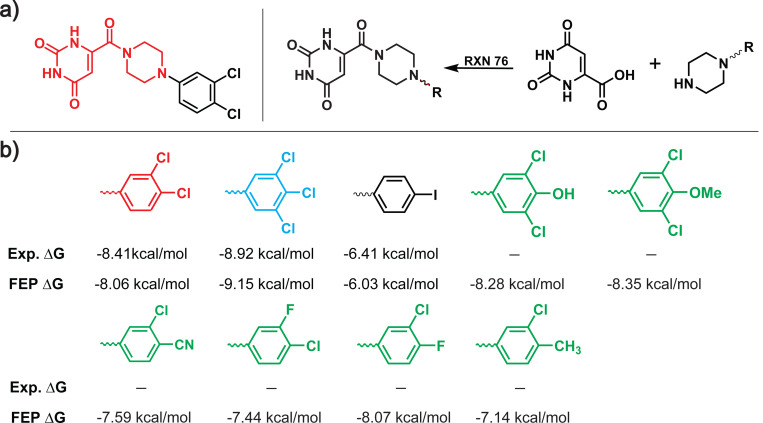
Hit expansion of binders to SARS-CoV-2 Mpro (PDB: 7LTJ) with SynLlama. (a). Target hit molecule and the predicted synthetic pathway to generate analogs of the target binder proposed by SynLlama. The core scaffold of the hit molecules are colored in red. (b). Hit compound and proposed analogs from SynLlama. The red-colored fragment represents the hit compound. The blue-colored fragment represents one SynLlama-generated analog that was also experimentally verified to have better binding affinity than the hit molecule. The green-colored fragments represent six potential binders that are within the 1 kcal/mol uncertainty range of the FEP score of the original hit.

As shown in Figure 3(a), the hit molecule has a core scaffold of uracil and ortho-dichlorobenzene connected by a piperizine linker. Inspired by an experimental hit expansion campaign by Kneller et al.^70^, we follow their practices to propose only functional group substitutions on the benzene ring while keeping the linker and uracil intact. Specifically, we use SynLlama to generate 50 synthesizable analogs constrained to only Enamine BBs of the hit compound and filter for molecules that only modifies the benzene ring, harvesting a total of 8 analog molecules that fulfill the criteria. The analogs are placed in a pose configuration similar to the original hit molecule and we perform FEP on all 8 molecules.

To verify the FEP results, we first choose around 10 synthesized and experimentally tested molecules to benchmark the accuracy of FEP for this system. In Figure S4, all calculated FEP values show a good correlation with experimental ΔG converted from IC50, with an average RMSE of less than 1*kcal/mol*. After this validation, we run FEP for the eight proposed molecules to assess their binding affinities. As shown Figure 3(b), SynLlama rediscovers two molecules from the reported hit expansion campaign, both with experimentally validated IC50 values. Notably, one molecule highlighted in blue was identified as the best compound from the expansion efforts reported by Kneller et al.^70^. The other six molecules highlighted in green also demonstrate desirable FEP-calculated binding affinities compared to the lead molecule, suggesting new directions for hit optimization in this ligand series. These results show that SynLlama effectively explores local chemistry with purchasable building blocks, saving time and effort for medicinal chemists who would otherwise manually enumerate and check availability at different steps.

## 4 Discussion

Motivated by recent advances in LLMs for chemistry^38,52–54^, we aim to leverage data-efficient supervised fine-tuning (SFT) to transform the general-purpose Meta Llama 3 into SynLlama, an LLM-based generator capable of proposing synthesizable molecules and deducing synthetic routes for target molecules or their close analogs. Throughout the study, we successfully show that SynLlama can effectively explore a custom-defined chemical search space composed of around 230,000 Enamine building blocks (BBs) and well-validated organic reactions (RXNs), after it has been fine-tuned on synthetic pathway data sampled from this specified chemical space. What’s more, despite utilizing nearly two orders of magnitude fewer synthetic pathways, SynLlama exhibits strong performance in key drug discovery tasks compared to existing models. Specifically, we have demonstrated that SynLlama can effectively aid in various stages of drug discovery that include synthesis planning, synthesizable analog generation for *de novo* molecules, and local hit expansion.

Because SynLlama is built on a general-purpose LLM instead of training from scratch^48–50^, it offers a number of unique advantages and possibilities for further improvement. For example, when generating our fine-tuning data, we sampled from the predefined chemical search space of 230K Enamine building blocks (BBs) and two sets of reaction templates (RXNs), but we did not embed these extensive requirements in the context window of the LLM. As a result, for our largest dataset with 2 million synthetic pathways, the model only saw each BB a few dozen times, while each RXN template appeared hundreds of thousands of times. Consequently, while SynLlama efficiently memorizes the allowed RXNs, it only captures the distribution of Enamine BBs, which enables SynLlama to extrapolate to unseen yet purchasable building blocks outside of Enamine. This generative ability surpasses other existing methods, which typically formulate the synthetic pathway generation as a Markov decision process within a defined chemical space.

In addition to its ability to extrapolate outside the training chemical space, the underlying Llama-3.2–1B used by SynLlama is relatively small and more predictive power would be expected if we train on larger LLMs with more data and compute power. However, we observe that a smaller LLM with fewer parameters can be turned into an expert model for complex tasks after SFT with sufficient data. This opens up opportunities to employ smaller expert models for various chemical tasks, benefiting from faster inference speeds, which can make these models more desirable. Moreover, optimal hyperparameters like temperature and top-p can vary between training and inference phases, depending on the downstream tasks. During inference, most valid raw outputs are generated under relatively low temperature and top-p settings. However, when the model is paired with reconstruction algorithms that require less strict adherence to reaction chemistry, higher temperature and top-p values can be used. This allows for a broader exploration of the Enamine chemical space, enabling the generation of more diverse and relevant analogs. This property is especially desirable in tasks that require extensive exploration, such as the hit expansion example we demonstrate. Another exciting success is that when SynLlama is paired with another generative model, the generated analogs can maintain docking scores while exhibiting a shift in synthetic accessibility scores. This result suggests that SynLlama can serve effectively as a post-processor for other *de novo* generative models, ensuring the production of more synthesizable compounds with clear reaction pathways.

Among the numerous opportunities that LLMs bring to the field of drug discovery, their natural language capabilities and recent advancements in reasoning are the most exciting features that allow users without coding expertise to interact directly with the models, effectively bridging the gap between computational methods and experimental research. We envision that expert users can employ prompt engineering and fine-tuning data to incorporate more realistic factors than those explored here. For instance, medicinal chemists could fine-tune LLMs within this generalizable SFT framework with building blocks and reaction templates of their own choice. In addition, they can consider synthesis cost, reaction conditions, improved selectivity, and protection factors at specific reaction steps for more detailed and powerful synthesis planning. We see our work as an initial attempt to demonstrate the effectiveness of LLMs in real experimental research, encouraging further studies for better utilization of these models.

## Supplementary Material

Supplement 1

## Figures and Tables

**Table 1:** Benchmarks of SynLlama inferences on 1000 training, testing, and ChEMBL data. We first select 1000 SMILES strings from the training examples, testing examples and the ChEMBL dataset, and then run inferences using SynLlama models trained on RXN Set 1. Benchmarking result for SynLlama models trained on RXN Set 2 can be found in Table S2. The detailed descriptions of each benchmark can be found in the main text. Here, we run SynLlama inferences at *T* = 0.1 and *TopP* = 0.1 to generate reproducible benchmarking results (see Supplementary Table S1).

		Model Config			
Dataset	Category	8B-100k	8B-500k	1B-500k	1B-2M

Training Data	Valid JSON	96.20%	97.20%	96.60%	98.00%
	Template Mem.	99.95%	100.0%	100.0%	100.0%
	BB Selection	99.80%	100.0%	99.72%	99.96%

	Valid SMILES	94.74%	99.53%	95.17%	99.46%
	Matched Reactants	78.62%	96.42%	80.19%	97.64%
	Good Products	78.34%	96.58%	81.26%	98.58%

Testing Data	Valid JSON	91.00%	94.20%	88.70%	93.90%
	Template Mem.	99.91%	100.0%	99.97%	100.0%
	BB Selection	99.75%	99.96%	99.73%	99.98%

	Valid SMILES	94.37%	99.13%	87.66%	99.50%
	Matched Reactants	77.51%	94.83%	65.63%	96.90%
	Good Products	74.11%	94.16%	69.54%	96.39%

ChEMBL Data	Valid JSON	98.80%	99.00%	99.20%	99.00%
	Template Mem.	99.90%	99.82%	99.37%	99.82%
	BB Selection	99.57%	99.23%	99.50%	99.47%

	Valid SMILES	92.02 %	96.38%	95.86%	95.23%
	Matched Reactants	54.52%	69.25%	64.62%	70.93%
	Good Products	67.69%	85.03%	75.81%	87.02%

**Table 2:** Comparison of synthesis planning performance among different methods. Both Testing, Enamine, and ChEMBL data sets are comprised of 1000 unseen molecules each. Details of each benchmark are described in the main text. The Morgan similarity scores include all analog molecules with successful synthesis pathways, as well as successfully reconstructed target molecules. The number of total training data and reaction set each method used for is: SynNet (**200K**, RXN Set 1) ChemProjector (**128M**, RXN Set 1), Synformer (**85M**, RXN Set 2), and SynLlama (**2M**, RXN Set 1 & 2). Inference details and identification of purchasable New BBs with Molport ^63^ are described in the Supplementary Information.

Dataset	Method	# of Recon. Mol.			Morgan Sim.
		Enamine BB	New BB	Total	

Testing-Set 1	SynNet^48^	107	−	107	0.46
	ChemProjector^49^	301	−	301	0.79

	SynLlama(RXN Set 1)	631	125	669	0.93

Testing-Set 2	SynLlama(RXN Set 2)	538	114	582	0.91

Enamine Data	SynNet	110	−	110	0.57
	ChemProjector	462	−	462	0.82
	Synformer^50^	660	−	660	0.91

	SynLlama(RXN Set 1)	527	100	568	0.87
	SynLlama(RXN Set 2)	691	232	741	0.92

ChEMBL Data	SynNet	54	−	54	0.43
	ChemProjector	133	−	133	0.60
	Synformer	198	−	198	0.67

	SynLlama(RXN Set 1)	165	95	223	0.66
	SynLlama(RXN Set 2)	197	152	287	0.68

## Data Availability

All the codes and data for SynLlama workflow are provided in a public accessible GitHub repository: https://github.com/THGLab/SynLlama under MIT License.
