# Supplementary material for: SynLlama: Generating Synthesizable Molecules and Their Analogs with Large Language Models
Source: ArXiv. 2025 Apr 18:arXiv:2503.12602v3. Preprint. [Version 3] (PMC12047903)
Supplement: Supplement 1 [file NIHPP2503.12602v3-supplement-1.pdf]

arXiv:2503.12602v3 [cs.LG] 18 Apr 2025

Supplementary Information SynLlama: Generating Synthesizable Molecules and Their Analogs with Large Language Models

Kunyang Sun[1], Dorian Bagni[1,Δ], Joseph M. Cavanagh[1,Δ], Yingze Wang[1], Jacob M. Sawyer[4], Andrew Gritsevskiy[5], Oufan Zhang[1], Teresa Head-Gordon*[1−3]

[1]Kenneth S. Pitzer Theory Center and Department of Chemistry, [2]Department of Bioengineering, [3]Department of Chemical and Biomolecular Engineering, University of California, Berkeley, CA, 94720 USA
[4]Department of Chemistry, University of Minnesota, 207 Pleasant Street SE, Minneapolis, MN 55455, USA [5]Contramont Research, San Francisco, CA, 94158 USA
[5]Contramont Research, San Francisco, CA, 94158 USA
[Δ]authors contributed equally
corresponding author: thg@berkeley.edu

Additional Methodology Details

Supervised Fine Tuning protocol. After preparing the reaction data and prompt-response pairs from the training chemical space, we fine-tune Llama-3.1-8B (8 Billion parameters) and Llama-3.2-1B (1 Billion parameters) using the Axolotl package[56?] . LLMs with more parameters require more resources to train and use, but they also typically perform better on a variety of tasks, which we consider in Results. For our SFT approach, we apply Low-Rank Adaptation (LoRA) with a rank of $r = 32$ and $\alpha = 16$ to the linear layers of the model.[?] We use FlashAttention-2[?] , with the Adam optimizer[?] , cross-entropy loss, and a cosine learning rate scheduler with a maximum learning rate of 2×10^{-5}.

Forward synthesis using Enamine BBs. When searching for the nearest neighbors of BBs, a natural choice is to perform a string-level similarity search based on SMILES strings, as this is the native format of SynLlama responses. For each RXN template, we systematically process all SMILES strings of its compatible building blocks that can participate in the reaction. First, we extract the full vocabulary of SMILES tokens and generate an n-gram representation by considering all possible consecutive token pairs (bigrams) and triplets (trigrams). Next, we identify the 1024 most frequently occurring n-grams across SMILES strings of all compatible BBs to form a representative token set for each individual RXN template. To facilitate efficient retrieval, we structure search trees based on the

term frequency-inverse document frequency (TF-IDF) scores[?] of these n-grams, prioritizing highly informative substructures and accelerating inference. Consequently, when a new SMILES string of the predicted BB is introduced, it can be efficiently processed through the tree, yielding a list of the top K matching SMILES strings.

In addition, Gao et al.[48] investigated using Morgan fingerprints[64], a molecular representation capturing local chemical environment, to search for the nearest neighbors of BBs based on their Tanimoto similarity[?] . Similarly to that stated above, for each RXN template, we also build a separate search tree for all compatible Enamine BBs using 256-bit Morgan fingerprint representation with a searching radius of 2. Our empirical observations indicate that combining the top K molecules from both the SMILES and Morgan fingerprint methods offers better performance than relying on the top 2K molecules from a single method. However, since we are working with an LLM model, the generated SMILES strings still have a small chance of being invalid, which prevents us from calculating their Morgan fingerprints. Therefore, we employ the both combined TF-IDF and Morgan fingerprint search trees when dealing with valid molecules, and revert to only a SMILES-based search when the generated SMILES strings are invalid.

LLM Inference Hyperparameters for Various Tasks. A key advantage of SynLlama, and LLMs in general, is their sensitivity to variations in hyperparameters, such as temperature (T) and top-p ($TopP$), which can significantly impact the performance of reconstruction and analog similarity. As shown in Supplementary Figure S2, SynLlama's raw outputs exhibit enhanced reaction chemistry comprehension when inferences are run at lower $TopP$ and within a reasonable range of T for both test sets. This configuration allows SynLlama to explore purchasable building blocks outside the Enamine library while maintaining synthesis validity. Conversely, increasing T and $TopP$ generally reduces SynLlama's ability to generate valid syntheses in its raw outputs. However, as Supplementary Figure S2 also illustrates, inferring with higher T and $TopP$ values than the optimal settings in raw outputs often leads to better overall average maximum similarity scores for reconstruction with Enamine BBs along. Nonetheless, excessively high settings can increase the failure rate.

Based on empirical observations, we recommend specific combinations of T and $TopP$ that effectively span a broad spectrum of tasks. These combinations optimize SynLlama's performance by balancing exploration and precision during inference.

- **Frozen**: $T = 0.1, TopP = 0.1$, repeated once. This setting prioritizes deterministic generation, ensuring minimal variability and high reproducibility.
- **Low**: $T = 0.6, TopP = 0.5$, repeated multiple times. This configuration allows for limited exploration while maintaining a degree of precision.
- **Medium**: $T = 1.0, TopP = 0.7$, repeated multiple times. This setting balances exploration and diversity, generating outputs with moderate randomness.
- **High**: $T = 1.5, TopP = 0.9$, repeated multiple times. This configuration promotes high diversity and creativity in generation but may introduce more variability in results.

We define different sampling strategies based on these core settings:

- **Frugal Sampling**: A total of 4 inferences.
  - $T = 0.1, TopP = 0.1$, repeated one time.
  - $T = 0.6, TopP = 0.5$, repeated one time.
  - $T = 1.0, TopP = 0.7$, repeated one time.
  - $T = 1.5, TopP = 0.9$, repeated one time.
- **Greedy Sampling**: A total of 10 inferences.
  - $T = 0.1, TopP = 0.1$, repeated one time.
  - $T = 0.6, TopP = 0.5$, repeated two times.
  - $T = 1.0, TopP = 0.7$, repeated three times.
  - $T = 1.5, TopP = 0.9$, repeated four times.
- **Frozen Only**: A total of 1 inference.
  - $T = 0.1, TopP = 0.1$, repeated one time.
- **Low Only**: A total of 5 inferences.
  - $T = 0.6, TopP = 0.5$, repeated five times.
- **Medium Only**: A total of 5 inferences.
  - $T = 1.0, TopP = 0.7$, repeated five times.
- **High Only**: A total of 5 inferences.
  - $T = 1.5, TopP = 0.9$, repeated five times.

Checking Commercial Availability of Building Blocks via Molport. In Results, we used the Molport platform to check whether a predicted BB is commercially available or not. Initially, we compiled a list of building blocks for searching and used the 'List Search' tab in the Molport website (https://www.molport.com/shop/swl-step-1) to check their availability. Once the SMILES strings were entered into the search interface, we set the search criteria to a minimum acceptable quantity of 500 mg and match types restricted to 'Exact' and 'Perfect' to search in the database of 'screening compounds' and 'building blocks.' Once the search completed, we downloaded the excel file under the 'Selected Items' column from the List Search result tab (https://www.molport.com/shop/swl-requests), which contained both the commercially available compounds and information about the supplying vendors.

Calculation of SA Scores. We calculate SA scores for both the iMiner-proposed molecules and SynLlama-generated analogs using the oracle functions named 'SA' implemented in the TDC Commons package[?] .

iMiner-Generated Molecules and Docking Procedures for Analogs. The iMiner algorithm[30], an 1D string-based LSTM model for SELFIES[?] string generation, was employed in this study. The molecules generated by iMiner were optimized using a composite

objective function comprising the AutoDock Vina docking score against the main protease SARS-CoV-2 (Mpro) and a custom-defined druglikeness score.

For molecular docking tasks, we obtained the SARS-CoV-2 Mpro crystal structure (PDB ID: 7L11[68]) from the Protein Data Bank[?] and processed it with PDBFixer[?] to add missing hydrogens and remove heteroatoms. The docking grid was centered at the geometric center of the ligand (XF1) from the corresponding PDB file ([x = -22, y = -4, z = -28]) using a cubic box with 20 Å sides. Both proteins and ligands were converted to PDBQT format using Meeko (https://github.com/forlilab/meeko). Docking was performed with AutoDock Vina using an exhaustiveness parameter of 64, and the best pose for each ligand was recorded. This protocol was consistently applied during both iMiner training and analog docking assessments.

The custom drug-likeness score is a composite score that evaluates 13 key molecular properties derived from the ChEMBL database. These properties capture both basic structural features and nuanced physicochemical characteristics, including the fraction of sp^3-hybridized carbons, the total number of heavy atoms, and the fraction of non-carbon atoms within these heavy atoms. Additionally, the score accounts for the counts of hydrogen bond donors and acceptors, the number of rotatable bonds, and the balance between aliphatic and aromatic rings, along with molecular weight. Complementing these are parameters such as the approximate log partition coefficient (alogP), polarizable surface area (PSA), the number of structural alerts, and the size of the largest ring present in the molecule. Each property contributes to the overall score through a weight that is inversely proportional to the entropy of its distribution in the ChEMBL database: properties with narrower and more informative distributions exert a stronger influence. By summing the log likelihoods of these properties with their respective weights, the score effectively biases the generative model to produce molecules that closely mimic the drug-like profiles observed in established therapeutics, ensuring that the exploration of chemical space remains focused on compounds with favorable bio-availability and efficacy profiles.

Free Energy Perturbation (FEP) Protocols. The relative binding free energies are calculated using GPU-accelerated AMBER22[?] (`pmemd.cuda.MPI`). AMBER14SB[?] and OpenFF-2.1.0[?] were used to parametrize the protein and the ligand, respectively. The protein structure (PDB code: 7LTJ) was downloaded from RCSB PDB and prepared with PDBFixer[?] to assign side-chain protonation states at pH=7.4 and add hydrogens. H163 was manually set to be its variant HIE (hydrogen added on Nϵ) to ensure the correct hydrogen bonding with the ligand. A sub-module `app.Modeller` in OpenMM[?] was used to immerse the protein-ligand complexes and unbound ligands in a cubic water box with 15Å buffer size and add ions (Na^+, Cl^-) to neutralize the system and maintain 0.15M ionic strength.

We used 16 unevenly distributed lambdas (0.0, 0.174, 0.226, 0.265, 0.330, 0.383, 0.432, 0.477, 0.522, 0.568, 0.617, 0.670, 0.735, 0.774, 0.826, 1.0) to transform the initial state to the final state in the free energy. This lambda settings was designed to maximize the phase space overlap between adjacent states with the second-order smooth-step function introduced. The transformations were performed with the modified SSC(2) softcore potentials ($m = n = 2, \alpha_{\mathrm{LJ}} = 0.5, \alpha_{\mathrm{Coul}} = 1$)[?] . Kartograf[?] algorithm was used to determine the common core region (SC) and soft core region (SC) atoms.

Each lambda state was subjected to the following simulation protocol to equilibrate the

system: (1) energy minimization without any constriants; (2) heating from 0 to 100 K at constant volume and temperature (NVT) ensemble over 20 ps, followed by MD at constant pressure and temperature (NPT) ensemble at 100 K for 20 ps; (3) heating to 200 K at NVT ensemble over 20 ps followed by another 20 ps at NPT ensemble at 200 K; (4) heating to 298.15 K at NVT ensemble over 20 ps followed by another 20 ps at NPT ensemble at 298.15 K; (5) another pre-prodcution equilibrium run at NPT ensemble for 500 ps. During the equilibration steps 2-4, restraints ($5\ \mathrm{kJ} \cdot \mathrm{mol}^{-1} \cdot \mathrm{Å}^2$) were applied to heavy atoms on the solute. Finally, a 5-ns production run was performed for each lambda state with the ACES enhanced sampling method[?] and replica exchange was attempted every 0.5 ps. All the simulations employed 4 fs time step with the mass of solute hydrogens repartitioned to 3 amu[?]. MBAR algorithm implemented in `alchemlyb`[?] was used to estimate the free energy change between two states and yield $\Delta\Delta G$. Then, the maximum likelihood estimation (MLE) method[?] was used to calculate the absolute binding free energy (ΔG) of each ligand and the ΔG was shifted to make the average of calculated ΔG of the ligands equal to the average of their experimental ΔG:

$$\sum_i \Delta G^{(i)}_{\mathrm{pred}} = \sum_i \Delta G^{(i)}_{\mathrm{expt}} = \sum_i RT \ln \mathrm{IC_{50}}^{(i)}$$

Supporting Tables

Task	Sampling Method	K	N_{Syn}
LLM Benchmark	Frozen Only	5	25
Synthesis Planning	Greedy Sampling	5	25
Synthesizable Analog	High Only	10	50
Hit Expansion	High Only	20	100

Table S1: Hyperparameters used for each task. Here, under each task name, we include the sampling method used for SynLlama inferences as defined in Additional Methodology Details. K represents the number of most similar SMILES string to take during the reconstruction algorithm. N_{Syn} represents the maximum number of synthesis routes to be tracked for each single SynLlama inference during the reconstruction algorithm.

Dataset	Category	SynLlama(RXN Set 1)	SynLlama(RXN Set 2)
Training Data	Valid JSON	98.00%	98.20%
	Template Mem.	100.0%	100.0%
	BB Selection	99.96%	99.96%
	Valid SMILES	99.46%	99.70%
	Matched Reactants	97.64%	97.95%
	Good Products	98.58%	97.97%
Testing Data	Valid JSON	93.90%	94.60%
	Template Mem.	100.0%	100.0%
	BB Selection	99.66%	100.0%
	Valid SMILES	99.50%	99.46%
	Matched Reactants	96.90%	97.25%
	Good Products	96.39%	96.19%
ChEMBL Data	Valid JSON	99.00%	99.00%
	Template Mem.	99.82%	100.0%
	BB Selection	99.47%	99.81%
	Valid SMILES	95.23%	97.33%
	Matched Reactants	70.93%	84.02%
	Good Products	87.02%	87.65%

Table S2: Benchmarks of SynLlama inferences using SynLlama models trained with two sets of reaction templates. Here, both models are fine-tuned on Llama-3.2-1B model with 2M reaction data generate using the same set of training building blocks. We select 1000 molecules for each model: training and testing data are generated using their corresponding reaction templates; ChEMBL data is the same set of 1000 molecules as described in the main text. All SynLlama inferences are run at $T = 0.1$ and $TopP = 0.1$.

Dataset	% of BB in Enamine	# of Raw Reconstructed Mol.		
		Enamine BBs	New BBs	Total
Testing	75.85%	506	125	563
Enamine	73.51%	510	100	557
ChEMBL	48.07%	161	95	221

Table S3: Comparison of Enamine BB presence and reconstruction with purchasable BBs across datasets at greedy temperature and top-p combo when using 91 RXN templates (RXN Set 1).

Dataset	% of BB in Enamine	# of Raw Reconstructed Mol.		
		Enamine BBs	New BBs	Total
Testing	76.61%	465	114	520
Enamine	68.34%	647	232	711
ChEMBL	48.04%	179	152	280

Table S4: Comparison of Enamine BB presence and reconstruction with purchasable BBs across datasets at greedy temperature and top-p combo when using 115 RXN templates (RXN Set 2).

Dataset	Method	Morgan	Scaffold	Gobbi
Testing-Set 1	SynNet[48]	0.46	0.51	0.35
	ChemProjector[49]	0.79	0.78	0.86
	SynLlama(RXN Set 1)	0.93	0.93	0.95
Testing-Set 2	SynLlama(RXN Set 2)	0.91	0.91	0.94
Enamine Data	SynNet	0.57	0.57	0.52
	ChemProjector	0.82	0.85	0.83
	Synformer[50]	0.91	0.92	0.89
	SynLlama(RXN Set 1)	0.87	0.88	0.85
	SynLlama(RXN Set 2)	0.92	0.94	0.92
ChEMBL Data	SynNet	0.43	0.20	0.27
	ChemProjector	0.60	0.59	0.56
	Synformer	0.67	0.72	0.72
	SynLlama(RXN Set 1)	0.66	0.67	0.63
	SynLlama(RXN Set 2)	0.68	0.69	0.66

Table S5: Similarity comparison across datasets and methods using Morgan, Scaffold, and Gobbi similarity scores. SynNet and Chemprojector are trained using RXN Set 1, and Synformer is trained using RXN Set 2. Scores are computed over all successfully synthesized analogs and reconstructed targets from Table 2.

Dataset	Method	Similarity		
		Morgan	Scaffold	Gobbi
Testing-Set 1	SynNet[48]	0.38	0.44	0.25
	ChemProjector[49]	0.68	0.68	0.80
	SynLlama(RXN Set 1)	0.78	0.79	0.85
Testing-Set 2	SynLlama(RXN Set 2)	0.78	0.79	0.85
Enamine Data	SynNet	0.51	0.51	0.45
	ChemProjector	0.67	0.72	0.69
	Synformer[50]	0.74	0.76	0.69
	SynLlama(RXN Set 1)	0.69	0.72	0.65
	SynLlama(RXN Set 2)	0.69	0.75	0.70
ChEMBL Data	SynNet	0.39	0.38	0.22
	ChemProjector	0.54	0.52	0.49
	Synformer	0.59	0.65	0.65
	SynLlama(RXN Set 1)	0.56	0.57	0.51
	SynLlama(RXN Set 2)	0.54	0.56	0.52

Table S6: Similarity comparison across datasets and methods using Morgan, Scaffold, and Gobbi similarity scores. SynNet and Chemprojector are trained using RXN Set 1, and Synformer is trained using RXN Set 2. The similarity metrics reported here are only for molecules that could not be fully reconstructed from Table 2.

Supporting Figures

**Instruction**	You are an expert synthetic organic chemist. Your task is to design a synthesis pathway for a given target molecule using common and reliable reaction templates and building blocks. Follow these instructions:\n\n1. **Input the SMILES String:** Read in the SMILES string of the target molecule and identify common reaction templates that can be applied.\n\n2. **Decompose the Target Molecule:** Use the identified reaction templates to decompose the target molecule into different intermediates.\n\n3. **Check for Building Blocks:** For each intermediate:\n - Identify if it is a building block. If it is, wrap it in <bb> and </bb> tags and save it for later use.\n - If it is not a building block, apply additional reaction templates to further decompose it into building blocks.\n\n4. **Document Reactions:** For each reaction documented in the output, wrap the reaction template in <rxn> and </rxn> tags.\n\n5. **Repeat the Process:** Continue this process until all intermediates are decomposed into building blocks, and document each step clearly in a structured JSON format.
**Input**	Provide a synthetic pathway for this SMILES string:<br>Cn1ncc(-c2ccc3c(c2)CC(C(=O)NCCOCc2ccccc2Cl)C3)n1
**Output**	"{'reactions':<br>[<br>{'reaction_number': 1,<br>'reaction_template':<br>'<rxn>[Cl,OH,O-:3][C$(C(=O)([CX4,c])),C$([CH](=O)):2]=[O:4].[N$([NH2,NH<br>3+1]([CX4,c])),N$([NH]([CX4,c])([CX4,c])):6]>>[N+0:6]-[C:2]=[O:4]</rxn><br>',<br>'reactants': ['NCCOCc1ccccc1Cl',<br>'Cn1ncc(-c2ccc3c(c2)CC(C(=O)O)C3)n1'],<br>'product': 'Cn1ncc(-c2ccc3c(c2)CC(C(=O)NCCOCc2ccccc2Cl)C3)n1'},<br>{'reaction_number': 2,<br>'reaction_template':<br>'<rxn>[Br:1][c$(c(Br)),n$(n(Br)),o$(o(Br)),C$([CH](Br)(=C)):2].[C$(C(B)<br>([CX4])([CX4])([CX4])),C$([CH](B)([CX4])([CX4])),C$([CH2](B)([CX4])),C$<br>([CH2](B)),C$(C(B)(=C)),c$(c(B)),o$(o(B)),n$(n(B)):3][B$(B([C,c,n,o])([<br>OH,$(OC)])([OH,$(OC)])),B$([B-1]([C,c,n,o])(N)([OH,$(OC)])([OH,$(OC)]))<br>:4]>>[C,c,n,o:2][C,c,n,o:3]</rxn>',<br>'reactants': ['O=C(O)C1Cc2ccc(Br)cc2C1',<br>'Cn1ncc(B2OC(C)(C)C(C)(C)O2)n1'],<br>'product': 'Cn1ncc(-c2ccc3c(c2)CC(C(=O)O)C3)n1'}<br>],<br>'building_blocks':<br>[<br>'<bb>NCCOCc1ccccc1Cl</bb>',<br>'<bb>O=C(O)C1Cc2ccc(Br)cc2C1</bb>',<br>'<bb>Cn1ncc(B2OC(C)(C)C(C)(C)O2)n1</bb>'<br>]<br>}"

Figure S1: Instruction, input, and output from the SynLlama model's inference on example SMILES string from Fig.1d. During data generation, all instructions remain the same, and the input-output pairs are generated within the training synthesizable chemical space. We enforce the JSON format in the output for our post processing algorithms. The output JSON has two parts: reactions and building blocks. In 'reactions', a series of reaction steps are generated, where the product of the next reaction serves as the reactant for the previous one. In 'building blocks', BBs are selected from the 'reaction' section and compiled into a list.

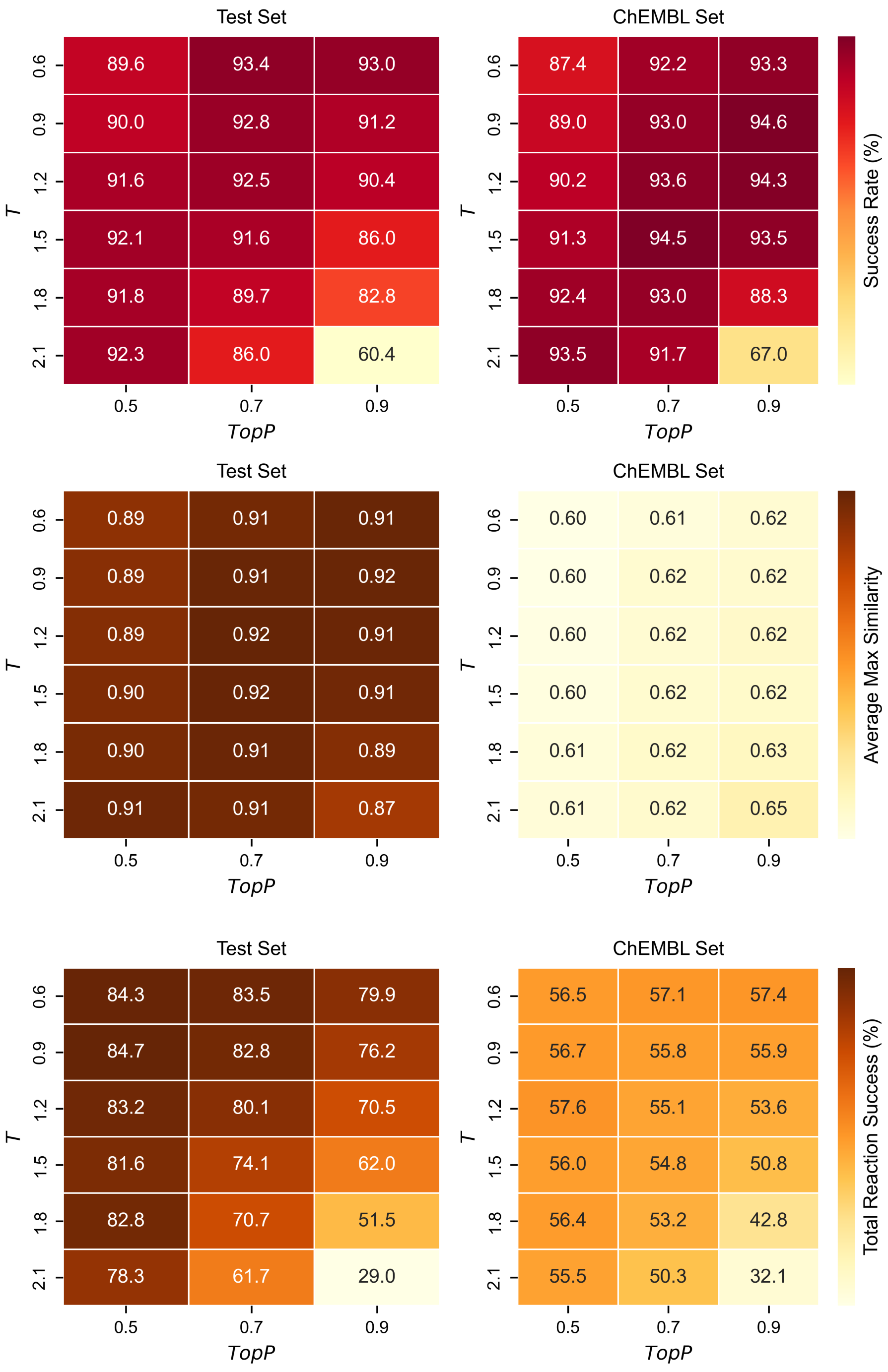

Figure S2: Reconstruction algorithm and SynLlama raw output benchmarks for SynLlama inferences on the Testing and ChEMBL sets under various temperature and top-p combinations. The first row represents the success rate of the Enamine reconstruction algorithm based on SynLlama inference outputs. The second row represents the average maximum Tanimoto similarity between the target and analogs generated via the reconstruction algorithm based on 4096-bit Morgan fingerprints. The last row represents the percentage of SynLlama raw outputs that can directly represent a retrosynthetic path for the input molecule without downstream processing with the reconstruction algorithm.

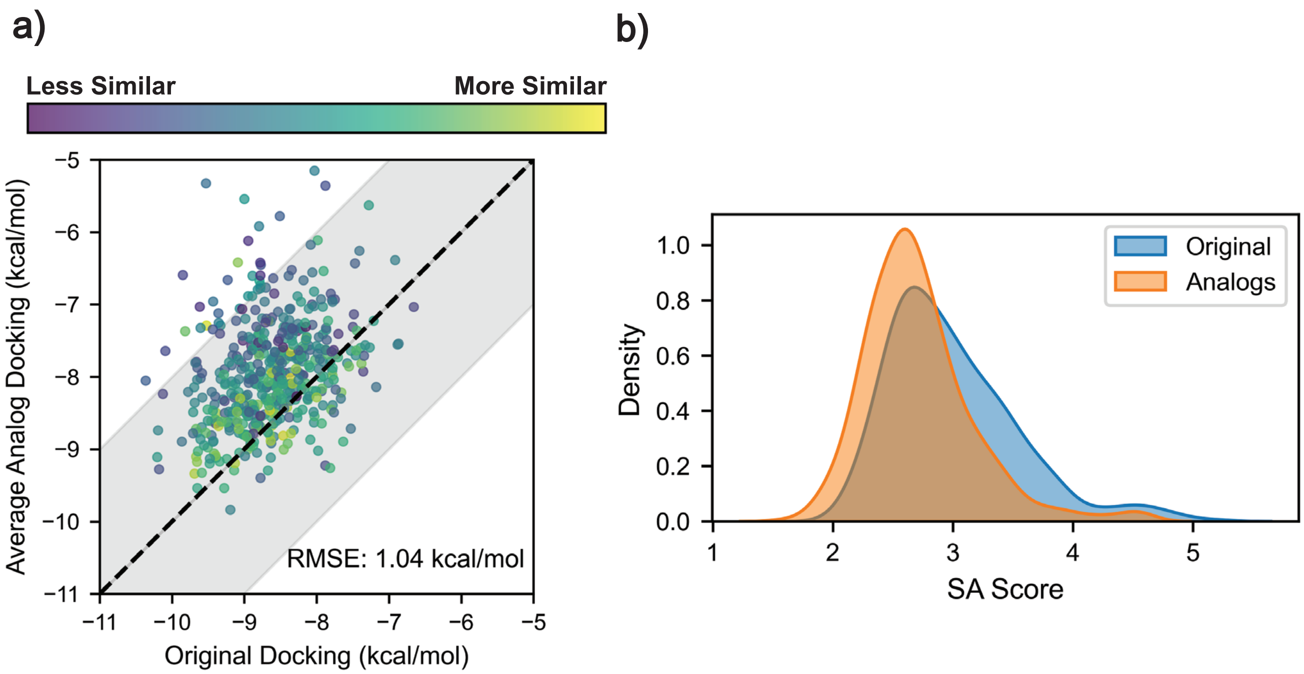

Figure S3: Docking score and SA score distribution between iMiner-generated molecules and proposed analogs from SynLlama model trained on RXN Set 1. (a). Correlation plot comparing docking scores of iMiner-generated molecules and the average docking scores of ten most similar analogs. Each data point is color-coded by the average Morgan fingerprint similarity computed between iMiner molecules and analogs, with brighter being more similar and darker being less similar. The shaded area represents an energy uncertainty range of $\pm 2 kcal/mol$, which is the typical uncertainty for AutoDock Vina scores. (b). SA score distribution of iMiner molecules and SynLlama-proposed analogs.

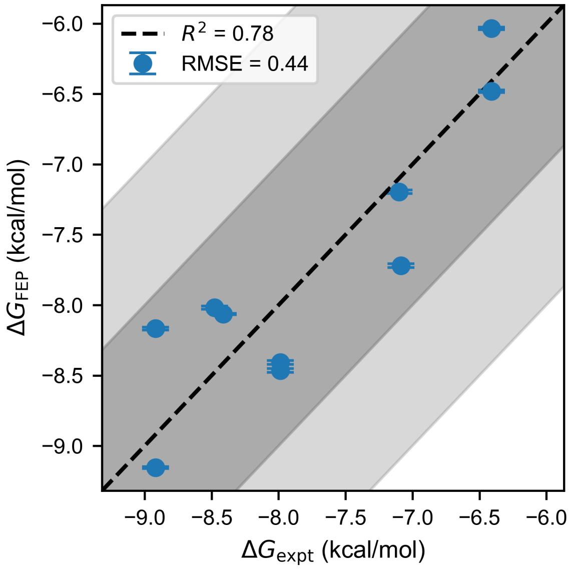

Figure S4: FEP benchmarking on the protein system 7LTJ. The experimental IC50 values extracted from Kneller et al.[70] are converted to the binding free energies, ΔG, which shows good correlations (RMSE $<$ 1 kcal/mol) with the ΔG calculated by FEP on the experimentally-tested molecules.
